# Items

**Published:** 1900-12

**Authors:** 


### ITEMS.

Messrs. Tarrant & Company, of New York, who have occupied the premises at the corner of Greenwich and Warren Streets in that city for nearly three-quarters of a century, suffered from a terrible disaster on the afternoon of Monday, October 29, 1900. First, an explosion occurred that blew up the magnificent Tarrant building together with several adjacent structures, after which fire broke out and completed the havoc, resulting in terrific destruction of life and property. The firm of Tarrant & Co. had so long and successfully conducted the drug business at the locality named that it had become one of the best known of the older established drug houses in New York.

Mr. Thomas F. Main, the president of the company, and his associates are entitled to receive the sympathy not only of their business confréres, but of the many physicians who have so long known this celebrated house that was renowned for its honorable dealings. We venture to hope that the business will soon be reëstablished on its former lines with an expanded plant.

---

Messrs. M. J. Breitenbach Company suffered the destruction of their building and plant in the disaster that overtook the Tarrant structure, above referred to. The building of this firm was an absolute wreck, not one portion remaining, but fortunately none of the employees of the company received the slightest injury—an almost miraculous escape under the circumstances. The Breitenbach Company have established themselves at 68 Murray Street, and are filling all their orders with their former promptitude. Their Leipzig house makes shipments twice a week and, fortunately, at the time of the calamity a cargo was in the custom house awaiting delivery. We tender both our sympathy and congratulations to this enterprising house.

---

On advertising page xxx. will be found an interesting opinion relating to antikamnia that was crowded out of its appropriate place.

---

The late Dr. W. E. Robbins, of Hamburg, N.Y., left a valuable physician's library which Mrs. Robbins offers for sale. It includes many standard and useful works such as the U. S. Dispensatory, Hartshorne's Essentials of Practical Medicine, Sajous's Annual of the Universal Medical Sciences, Dunglison's Medical Dictionary, together with treatises on obstetrics, surgery, internal medicine, the several specialties and works on miscellaneous subjects. For particulars address Mrs. W. E. Robbins, Hamburg, N.Y.

